# Free Radical Generation in Far-UV Synchrotron Radiation Circular Dichroism Assays—Protein and Buffer Composition Contribution

**DOI:** 10.3390/ijms222111325

**Published:** 2021-10-20

**Authors:** Paolo Ruzza, Claudia Honisch, Rohanah Hussain, Giuliano Siligardi

**Affiliations:** 1 Institute of Biomolecular Chemistry of CNR, Padua Unit, via Marzolo, 1, 35131 Padova, Italy; claudiahonisch@gmail.com; 2 Department of Chemical Sciences, University of Padua, via Marzolo, 1, 35131 Padova, Italy; 3 Diamond Light Source Ltd., Harwell Science and Innovation Campus, Didcot, Oxfordshire OX11 0DE, UK; rohanah.hussain@diamond.ac.uk

**Keywords:** photo-oxidation, spectroscopy and biochemistry buffers, protein denaturation, reactive oxygen species (ROS), synchrotron radiation circular dichroism (SRCD)

## Abstract

A useful tool to analyze the ligands and/or environmental contribution to protein stability is represented by the Synchrotron Radiation Circular Dichroism UV-denaturation assay that consists in the acquisition of several consecutive repeated far-UV SRCD spectra. Recently we demonstrated that the prevailing mechanism of this denaturation involves the generation of free radicals and reactive oxygen species (ROS). In this work, we analyzed the effect of buffering agents commonly used in spectroscopic measurements, including MOPS (3-(N-morpholino) propanesulfonic acid), HEPES (4-(2-hydroxyethyl)-1-piperazineethanesulfonic acid), TRIS-HCl (tris-hydroxymethil aminomethane hydrochloride), and phosphate, on the efficiency of protein denaturation caused by exposure to UV radiation. Fluorescence experiments confirmed the presence of ROS and were used to determine the rate of ROS generation. Our results indicate that the efficiency of the denaturation process is strongly influenced by the buffer composition with MOPS and HEPES acting also as scavengers and that the presence of proteins itself influenced the ROS formation rate.

## 1. Introduction

Circular dichroism (CD) spectroscopy is a valuable biophysical tool for studying the structure and conformation of biomolecules [1]. In particular, CD analysis in the far-UV region is widely used to evaluate the secondary structure of proteins/peptides in solution, as well as the influence of either ligands or environment (polarity, pH, ionic strength) on protein conformation and stability [2]. This analysis is often hampered by the buffer properties and the selection of the right buffer to use is a critical step. This is because it must preserve the activity and stability of the biological system as well as optimizing the experimental conditions by being transparent in the far-UV region.

The use of synchrotron radiation (SR) of third-generation synchrotrons as a light source has overcome the limitation of the lower photon flux of benchtop CD instruments extending the range of analysis in the vacuum-UV region. In particular, the highly collimated microbeam generated at B23 beamline of Diamond Light Source enables the measurements of CD spectra under a broad variety of conditions using: (i) capillaries with sub-milliliter volume of sample [3]; (ii) multiplate for high-throughput CD [4,5]; (iii) high pressure up to 200 MPa [6]; and (iv) CD imaging at high spatial resolution of 50 µm [7]. The high brilliance and photon flux of B23 [8] has been found to induce protein denaturation when repeated consecutive spectra were scanned. The rate of denaturation was found to be sensitive to protein folding, concentration, and environmental conditions [9], leading to the development of a novel method of protein analysis [10]. Of the different mechanisms proposed to elucidate the protein UV-photo denaturation, free radicals and reactive oxygen species (ROS) are the main one [11,12]. The main targets of far-UV (180–250 nm) and near-UV (250–320 nm) light irradiation of protein are the peptide amide bond, the aromatic and cysteine residues. The excitation of these targets is followed by distinct processes including fluorescence, triplet state formation, reaction with oxygen to form radicals, or excited state photochemical or photophysical processes such as photoionization [13]. In dilute aqueous solutions, at micromolar protein concentration, UV light interacts principally with the solvent water molecules (about 55 M) that are converted into highly reactive species such as hydroxyl radical HO^•^, hydrated electron e^−^_aq_, and hydrogen radical H^•^ (Scheme S1 in Supplementary Information) [14].

The protein UV-photo denaturation assay developed at B23 beamline requires a thorough investigation of the factors that can interfere in the analysis. In particular, much less is known regarding possible buffers and/or biopolymers interferences with studies on protein denaturation induced by exposure to UV radiation. Often these observations go unpublished unless they help the understanding of the systems being studied or their interferences with assays.

To overcome this lack of information, the efficiency of the SRCD UV-denaturation assay has been investigated for different proteins such as bovine fatty acid free (BSAff), pharmaceutical human serum albumin (HSA), lysozyme (HEWL), ubiquitin (Ubi), rabbit IgG (IgG) and poly-L-lysine (PLL) as representative model proteins. These proteins were dissolved in four buffer types typically used in biological and spectroscopic studies, such as MOPS, HEPES, TRIS-HCl, and phosphate (PB) (Figure S1 in Supplementary Information). Here, the UV-denaturation assays were performed acquiring 50 consecutive SRCD spectra in the 185–260 nm range (top-up mode ring current of 300 mA), while the presence of reactive oxygen species and their rate of generation were evaluated using the ROS-sensitive fluorescent probe dehydrorhodamine-123 (DHR-123) that under UV irradiation is converted to Rhodamine-123 (Rh-123) [15].

Our results indicated that the buffer composition influenced and affected the rate of both protein denaturation and free radical formation. It is important to note that the rate of DHR-123 conversion to Rh-123 increased in the presence of proteins.

## 2. Results and Discussion

The effect of UV irradiation on the secondary structure of protein has been evaluated collecting 50 consecutive SRCD spectra in the 185–260 nm range of protein solutions. The estimation of the secondary structure elements (α-Helix, β-Strands, Turns and Unordered) of BSAff, HSA and HEWL shows a high content in α-helix, while IgG and Ubi possess a predominant β-sheet secondary structure and the CD spectrum of PLL is consistent with a left-handed extended helix of poly-proline type II [16,17].

### 2.1. Effect of Light on Protein Structure

**Figure 1:**
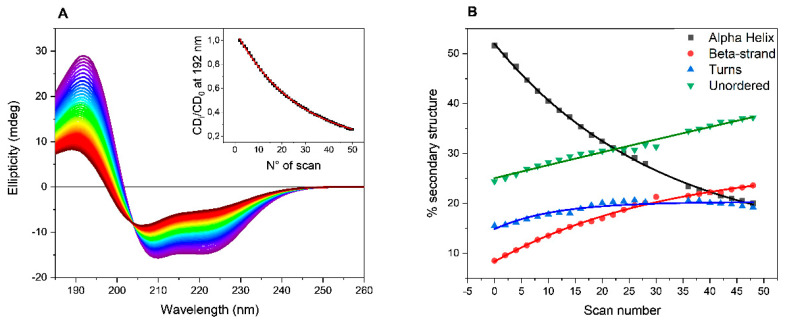
UV-denaturation experiments of BSAff. (**A**) 50 consecutive repeated scans of BSAff (6.8 µM) in 10 mM phosphate buffer, pH 7.00. (insert) Time-course of CD values at 192 nm versus scan numbers. (**B**) Secondary structure estimation of BSAff throughout the UV-denaturation experiment. Spectra were acquired in the 185-260 nm range at module B end station of beamline B23 at Diamond Light Source synchrotron facility, Harwell Science and Innovation Campus (Didcot, UK), using a 0.02 cm quartz cuvettes and 0.5 mg/mL (7.6 µM) protein concentration. Bandwidth was 1 nm, scan speed was 39 nm/min, and top-up mode ring current of 300 mA.

The collected SRCD spectra of BSAff in PB are reported as example in Figure 1A. A decrease in the intensity of both positive (192 nm) and negative (208 and 222 nm) bands was observed at increased number of scans indicating a progressive loss in the ordered structure from the estimation of the secondary structure content (SSE) using CONTNILL algorithm [18,19]. As shown in Figure 1B, after 50 consecutive scans, the α-helix content decreased from initially 51% to 22%, while the β-sheet content increased from 9% to 25%, accompanied by an increase in both turn (from 15 to 22%) and unordered (from 24 to 31%) elements of secondary structures content. Such behavior was observed repeating the UV-denaturation assays with BSAff dissolved in the other buffers such as HEPES, MOPS and TRIS-HCl (Figure S2 in Supplementary Information).

To quantify the efficiency of buffers to promote BSAff denaturation, the ellipticity values at 192 nm were plotted versus the number of scans that is proportional to the exposure time to UV light. In the experiment, each scan took 3 minutes, hence the total UV-denaturation assay was completed in 2 and half hours. The data can be fitted with a first-order exponential decay equation (insert of Figure 1A, and Figure S3 in Supplementary Information) and the decay rate (k = 1/t) as well as the half-life (t_1/2_) values are reported in Table 1. A rapid process is characterized by higher decay rate and lower half-life values.

As reported in Table 1, the higher decay rates were determined in both TRIS-HCl and PB buffer solutions (decay rate 0.039 and 0.037 min^−1^, respectively), while in MOPS and HEPES the lower values were detected (decay rate 0.031 and 0.028 min^−1^, respectively). 

Statistical tests using two sample t-test (OriginLab) was performed, and statistically significant differences (*p* < 0.05) were observed (Table S1 in Supplementary Information).

The low decay rate values and the increment in the half-life values determined in MOPS and HEPES buffer solutions may be explained with the capability of these molecules to interact with BSA, supporting the native structure of this protein. The interactions involved in this process essentially occur between the buffer molecules, MOPS and HEPES, and the hydration layers surrounding the peptide backbone of BSA protein [20]. Moreover, docking studies in gas phase showed that MOPS and HEPES molecules can bind to domains II and III (Figure S4 in Supplementary Information) of BSA [20]. Although MOPS and HEPES are structurally related compounds, slight differences in the ability to interact and stabilize the structure of BSA have been detected [20,21], consistent with the difference in the decay rate and half-life values determined in the UV-denaturation assays (Table 1).

A similar drastic change of the CD spectral shape of the investigated proteins of Table 1, HSA (pharmaceutical and dialyzed), IgG, Ubi, PLL and HEWL has been observed in all buffers after 50 consecutive SRCD scans (Figure S5 in Supplementary Information), with the exception of that for PLL. In this case, the CD shape remained unchanged with only a small decreased intensity of the diagnostic negative band at 197 and positive band at 215 nm. Collectively these data indicate that the exposure to UV radiation induces a loss of secondary structure more evident in highly structured proteins (Figure S6 in Supplementary Information).

The lowest decay rate values for these proteins were determined in both MOPS and HEPES buffer solutions. These data suggest the presence of a mechanism that reduces the UV-induced protein denaturation in MOPS and HEPES buffers. This is consistent with the recent observation that ROS can efficiently react with both MOPS and HEPES molecules generating morpholino/piperazino radicals that evolve into N-oxide. The latter, subtracting free radicals to the medium, behaves as radical scavenger, hence reducing the efficiency of the overall UV-denaturation process [22,23].

The decay rate values were also correlated with the net charge of proteins. For this purpose, the charge at pH 7.0 of proteins was determined starting from their sequences by the Prot pi Protein Toll web application and plotted versus the determined decay rate values (Figure S7 in Supplementary Information). The net charge has not been determined for IgG and PLL as the sequence and degree of polymerization, respectively, are unknown. As shown in Figure S7, the decay rate increased when increasing the net charge of protein suggesting that the UV-denaturation process is also correlated with the charge of protein that could influence the physicochemical properties of hydration water that plays different roles including the rate of transmission of ROS from the solvent phase to the protein surface.

### 2.2. Reactive Oxygen Species (ROS) Detection

Reactive oxygen species produced by UV radiation were detected by fluorescence spectroscopy using the positive fluorogenic probe DHR-123. This molecule is converted into the highly fluorescent rhodamine-123 (Rh-123) upon reaction with free radicals including the hydroxyl radical HO^•^ and the superoxide anion radical O_2_^•−^ (Scheme S2 in Supplementary Information). The fluorescence intensity at 524 nm of Rh-123 is proportional to the amount of oxidized DHR-123 and hence the rate of fluorescence increment is proportional to the rate of free radical generation. The irreversibility of DHR-123 oxidation has the advantage that transient events can produce permanent effects.

In these fluorescence studies, synchrotron radiation was replaced by a system consisting of four UV-C lamps emitting at 254 nm.

#### 2.2.1. Starting from Buffer

**Figure 2:**
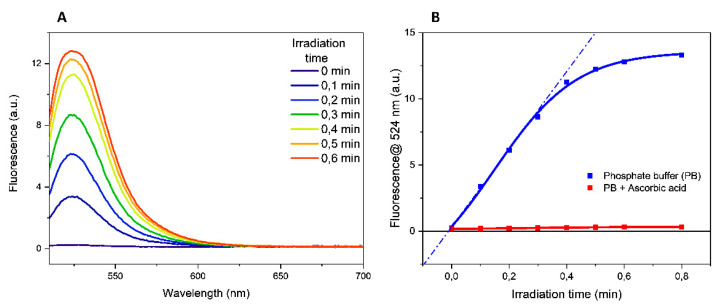
(**A**) Fluorescence emission spectra of oxidized DHR-123 in 10 mM phosphate buffer, pH 7.00. (**B**) Time-courses of fluorescence emission at 524 nm of oxidized DHR-123 in tested buffer solutions. A DMSO stock solution of DHR-123 was diluted in tested buffer obtaining a 3.17 µM solution (DMSO percentage less than 0.05%). Solutions were irradiated using UV-C lamps in a BioLink 254 photoreactor. Fluorescence spectra were recorded after excitation at 505 nm with a slit of 4 nm.

Experiments irradiating the buffers alone showed that the increment in the fluorescence emission at 524 nm was proportional to the irradiation time of the solution containing the fluorogenic probe DHR-123 (the fluorescence data obtained in PB were reported in Figure 2A as an example).

A quantitative evaluation of this process can be obtained determining the oxidation rate that corresponds to the slope of the tangent to the time-course curves (Figure 2B and Table 2). As reported in Table 2, in PB and TRIS-HCl buffer solutions the oxidation rate of DHR-123 has comparable and intermediate values, while the highest and the lower values were determined in MOPS and in HEPES buffer solutions, respectively.

To confirm that DHR-123 oxidation was mediated by free radicals, parallel experiments were performed in the presence of 0.1 mM ascorbic acid, a radical scavenger. No increase in the fluorescence emission was determined in the presence of scavenger, proving the involvement of free radicals in the probe oxidation. The time course in the presence of ascorbic acid in PB buffer is illustrated in Figure 2B. To rule out any thermal effects induced to the aqueous solution following the UV exposure as the cause of the DHR-123 conversion, fluorescence spectra were carried out in the 20–80 °C temperature range. The absence of any fluorescence emission (Figure S8 in Supplementary Information) clearly confirmed that the increased fluorescence emission upon UV irradiation was solely due to the formation of free radicals rather than thermal effects.

#### 2.2.2. Starting from Protein Solution

In the presence of proteins, the oxidation rate values were greater in all buffers as reported in Table 2. This increment could be due to electron and energy transfer reactions between excited proteins and molecular oxygen that produces more reactive oxygen species [11,13]. This is conceivable as proteins can directly absorb UV light either by side-chains chromophores such as Trp, Tyr, Phe and the disulfide bond or by the peptide bonds. Upon absorption of UV radiation, residues are converted to their excited singlet states that readily are converted to the triplet states with much longer lifetimes and able to undergo electron transfer to a nearby group and hydrogen abstraction [11,13]. Photolysis studies also revealed that the excited states can eject an electron to the solvent, yielding solvated electrons, e^−^_aq_, that react with O_2_ generating O_2_^•−^ radical [11,13].

**Figure 3:**
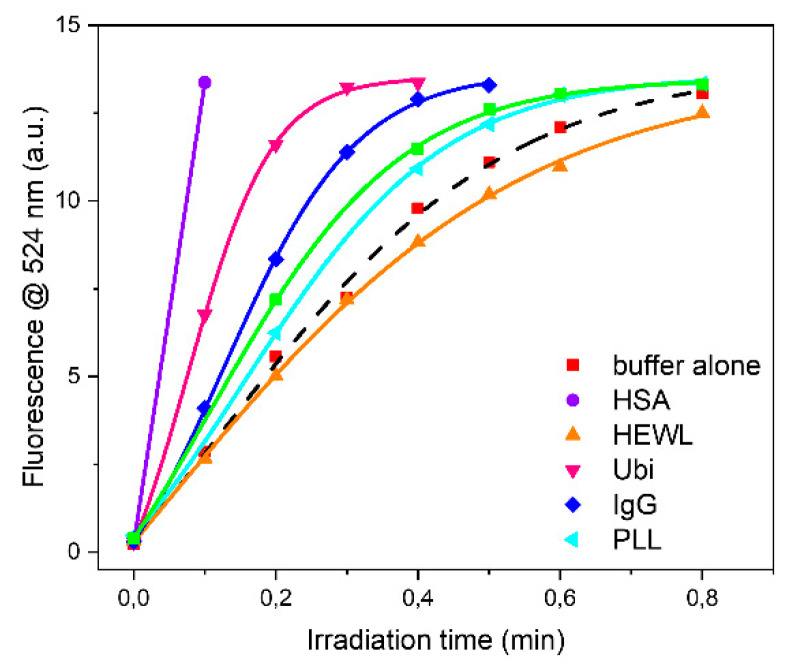
Time-courses of DHR-123 conversion in 10 mM HEPES buffer, pH 7.00, in the presence of different proteins. Solutions were irradiated using UV-C lamps in a BioLink 254 photoreactor. Fluorescence spectra were recorded after excitation at 505 nm with a bandwidth of 4 nm.

The higher oxidation rate observed in the investigated buffers with pharmaceutical HSA could also be due to the acetyl-tryptophan added as stabilizer in the pharmaceutical HSA formulation. To confirm this hypothesis, parallel experiments were performed on PB buffer solutions containing either dialyzed pharmaceutical HSA or acetyltryptophanamide (Ac-Trp-NH_2_). The oxidation rates of the dialyzed pharmaceutical HSA (13.8 a.u./min) were about 9-fold lower than the non-dialyzed (130.6 a.u./min) (Figure 3). On the other hand, the oxidation rate of Ac-Trp-NH_2_ was almost identical to that of pharmaceutical HSA (129.0 a.u./min and 130.6 a.u./min, respectively). These data clearly confirmed the involvement of the aromatic Trp residue in the ROS production, indicating this molecule as a strong pro-oxidant (Figure S9 in Supplementary Information).

Apart from HSA, ubiquitin and rabbit IgG were the proteins that affected more the oxidation rate of DHR-123. On the contrary, PLL was the least active. This is not a surprise as the dominant conformation is an extended left-handed helix not stabilized by intramolecular hydrogen bonds between NH and CO groups but rather with water molecules. BSAff and HEWL had very similar effects on the oxidation rate. 

**Figure 4:**
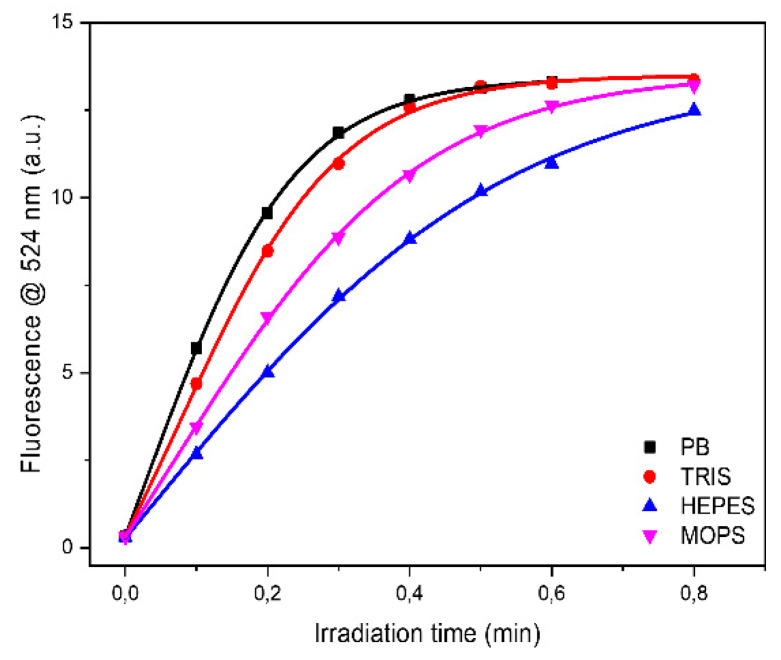
Time-courses of DHR-123 conversion in the presence of HEWL in different buffers. Solutions were irradiated using UV-C lamps in a BioLink 254 photoreactor. Fluorescence spectra were recorded after excitation at 505 nm with a bandwidth of 4 nm.

Analyzing each protein as a function of buffer it can be observed that the higher oxidation rate was observed in PB buffer for HEWL, IgG and Ubi respectively, or in TRIS buffer for BSAff, while the lower values were found in MOPS and in HEPES buffers (Figure 4 and Table 2). The rate of protein UV-photo denaturation as a function of protein formulation revealed the scavenger property of both MOPS and HEPES for the ROS formation.

## 3. Materials and Methods

Dihydrorhodamine 123 (DHR-123), bovine serum albumin fatty acid free (BSAff), hen egg white lysozyme (HEWL), rabbit IgG antibody, Ubiquitine (UBI), poly-L-Lys (PLL) and DMSO were purchased from Sigma-Aldrich (Milan, Italy) and used without any other treatment. Pharmaceutical human serum albumin (HSA) was purchased as commercially available human drug (Albital®, Kedrion, Lucca, Italy), which is stabilized with 16 mM Ac-Trp-OH, and was used as it is or after dialyzation to remove the stabilizer excess.

Fluorescence experiments were carried out using a Chirascan Plus CD Spectropolarimeter with fluorescence attachment (Applied Photophysics, Leatherhead, U.K.). Briefly, 1.5 μL of DHR-123 in DMSO (2 mg/mL) were added to 3000 μL of 10 mM buffer (PB, TRIS, HEPES or MOPS), pH 7.00, in a Suprasil fluorescence cell (Hellma analytics, Müllheim, Germany) with 1.0 cm path length and irradiated using a BioLink 254 photoreactor (Vilber, Eberhardzell, Germany) generating 0.120 J/cm^2^ radiation energy or 0.2 J/s. The CD spectra of the irradiated protein solutions with BioLink 254 nm lamp or with B23 beamline showed qualitatively the same change in spectral profile indicative of similar protein denaturation behavior. Quantitatively the 254 nm lamp provided a much higher radiation power than that of the B23 beamline inducing a much faster protein UV denaturation. The fluorescence emission spectrum of irradiated DHR-123 solution in the 510–700 nm range (Ex 505 nm, slit 4 nm) was recorded at different times. The emission spectra of the non-irradiated solution were recorded in a temperature range between 5 °C and 85 °C, with 10 °C steps, allowing 8 min equilibration.

The protein denaturation was monitored by synchrotron radiation circular dichroism (SRCD) spectroscopy collecting 50 repeated consecutive scans in the 185–260 nm region at module B end station of beamline B23 at Diamond Light Source synchrotron facility, Harwell Science and Innovation Campus (Didcot, UK) in a 0.02 cm Suprasil fused quartz cells, bandwidth of 1 nm, digital resolution of 0.5 nm. The collected data were analyzed with CDApps [19] and OriginPro2018 (OrigionLab Corporation, Northampton, MA, USA) software.

The rates of denaturation from SRCD data at 191 nm were calculated fitting the exponential decay equation in CDApps [19], or y = y_0_ + Ae^(−x/t)^ as ExpDec1 fit of OriginPro 2018 software where A = amplitude, t = time constant and y_0_ = offset. The derived parameters rate of denaturation k = 1/t (min^−1^). All kinetics results were reported with the associated standard deviation (SD), and the statistical significance of the data obtained was evaluated by two sample t-test run using OriginPro2018 software.

## 4. Conclusions

The protein UV-denaturation assay described in the manuscript has been used as a simple and faster method of assessing the relative UV-photo stability of proteins of the same mg/mL concentration, measured in the same pathlength cuvette cell, and under a variety of environmental conditions such as solvent composition and ligands.

The influence of both protein and buffer composition under a single UVC wavelength of 254 nm and far-UV (180–250 nm) synchrotron radiation spectral range was investigated using circular dichroism and fluorescence spectroscopies.

The stability of proteins was found to be affected by both protein folding and buffer composition. We have shown that proteins of dominant α-helix or β-sheet secondary structure are more affected by synchrotron radiation than poly-L-lysine adopting an extended left-handed helix of poly-proline type II at neutral pH. The protein denaturation rate was found to be much greater in both PB and TRIS than in MOPS and HEPES buffers suggesting a scavenging property for the latter one.

Fluorescence studies performed using the positive fluorogenic probe DHR-123 confirmed that the generation of reactive oxygen species (ROS) and rate of production by UV irradiation was affected by both buffer composition and protein folding.

This observation is more important not only in the design and evaluation of UV-denaturation assay but also for the food and pharmaceutical industry where UV light is applied as an alternative method of sterilization. The determination of the protein photo stability as a function of environment is an important property to be evaluated as the short exposure to intense UV light as well as prolonged exposure of lower levels of photon flux can induce a loss of structure leading to loss in protein activity and or function.

## Figures and Tables

**Table 1:** Decay rate and half-life values of UV denaturation of tested protein determined acquiring 50 consecutive repeated scans.

Buffer	PB		TRIS		MOPS		HEPES	
Protein	k min^−1^∙10^3^	t_1/2_ min	k min^−1^∙10^3^	t_1/2_ min	k min^−1^∙10^3^	t_1/2_ min	k min^−1^∙10^3^	t_1/2_ min
BSAff	37.3 ± 0.5	18.6 ± 0.3	39.2 ± 0.6	17.8 ± 0.2	31.4 ± 0.5	22 ± 0.4	28.1 ± 0.5	24.4 ± 0.4
HSA	25.1 ± 0.7	27.9 ± 0.7	24.3 ± 0.6	28.8 ± 0.6	20.2 ± 0.5	33.8 ± 0.8	18.4 ± 0.6	39.0 ± 1.0
IgG	16.3 ± 1.7	44.1 ± 0.9	17.2 ± 1.9	41.3 ± 0.9	13.2 ± 2.2	54.5 ± 1.4	11.1 ± 1.3	65.7 ± 0.9
Ubi	3.0 ± 0.7	209 ± 42	5.1 ± 1.1	130 ± 27	4.2 ± 0.6	188 ± 31	2.0 ± 0.6	292 ± 81
PLL	7.1 ± 0.9	93.2 ± 6.5	12.0 ± 0.4	59.7 ± 1.6	10.1 ± 0.7	68.0 ± 9.3	7.2 ± 0.4	101 ± 16
HEWL	28 ± 1.2	24.8 ± 0.9	31 ± 1.2	22.6 ± 0.8	21 ± 1.3	33.0 ± 1.9	23 ± 1.4	30.7 ± 1.9

**Table 2:** Oxidation rate and doubling time (dt) values of DHR-123 conversion to fluorescent Rh-123 in tested buffer solutions.

Buffer	PB		TRIS		HEPES		MOPS	
	Rate min^−1^∙10^3^	dt min	Rate min^−1^∙10^3^	dt min	Rate min^−1^∙10^3^	dt min	Rate min^−1^∙10^3^	dt min
alone	30.40 ± 0.08	0.21 ± 0.08	30.20 ± 0.06	0.21 ± 0.06	38.85 ± 0.07	0.16 ± 0.07	25.81 ± 0.06	0.25 ± 0.06
BSAff	38.53 ± 0.02	0.16 ± 0.02	43.37 ± 0.01	0.15 ± 0.01	33.22 ± 0.01	0.19 ± 0.01	35.41 ± 0.01	0.18 ± 0.01
HSA *	130.62	0.04	131.17	0.04	130.77	0.04	131.01	0.04
HEWL	48.91 ± 0.01	0.12 ± 0.01	43.56 ± 0.02	0.14 ± 0.02	31.42 ± 0.02	0.20 ± 0.02	24.15 ± 0.01	0.27 ± 0.01
IgG	56.65 ± 0.06	0.11 ± 0.06	53.46 ± 0.08	0.13 ± 0.08	42.26 ± 0.01	0.12 ± 0.01	43.91 ± 0.04	0.15 ± 0.04
Ubi	86.80 ± 0.50	0.03 ± 0.01	54.86 ± 0.83	0.06 ± 0.01	57.43 ± 0.02	0.09 ± 0.02	68.99 ± 0.02	0.09 ± 0.02
PLL	26.28 ± 0.04	0.23 ± 0.04	28.16 ± 0.02	0.22 ± 0.02	28.20 ± 0.01	0.22 ± 0.01	29.67 ± 0.04	0.21 ± 0.04

* The DHR-123 conversion in the presence of pharmaceutical HSA saturated immediately and the fluorescence data are fitted only between two points.

## Supplementary Materials

The following are available online at https://www.mdpi.com/article/10.3390/ijms222111325/s1. Figure S1: Structure of the investigated buffering agents. Figure S2: SRCD UV-denaturation experiments performed on Bovine Serum Albumin fatty acid free (BSAff) in the four investigated buffers. Figure S3: CDi/CD0 at 192 nm plotted versus the number of SRCD scans for Bovine Serum Albumin fatty acid free (BSAff). Figure S4: Domain structure of BSA. Figure S5: SRCD UV-denaturation experiments of Bovine Serum Albumin fatty acid free (BSAff), pharmaceutical human serum albumin (HSA), lysozyme (HEWL), ubiquitin (Ubi), rabbit IgG (IgG) and poly-L-lysine (PLL) in phosphate buffer. Figure S6: Change in secondary structure content determined for Bovine Serum Albumin fatty acid free (BSAff) pharmaceutical human serum albumin (HSA), lysozyme (HEWL), ubiquitin (Ubi), rabbit IgG (IgG) and poly-L-lysine (PLL) in all the investigated buffers after 50 consecutive SRCD spectra. Figure S7: Correlation between protein charge (z) and decay rate values of protein UV denaturation. Figure S8: Fluorescence emission spectra of DHR-123 when heated from 5 to 85 °C. Figure S9: Fluorescence emission spectra of oxidized DHR-123 in the presence of pharmaceutical Human Serum Albumin (HSA) or its dyalized solution. Scheme S1: UV-irradiation effects on water molecules. Scheme S2: Dihydrorhodamine-123 (DHR-123) oxidation. Table S1: statistical analysis on all proteins tabulated in Table 1 with all possible buffer pairs.

## References

[1] Dodero V.I., Quirolo Z.B., Sequeira M.A. (2011). Biomolecular studies by circular dichroism. Front. Biosci..

[2] Li C.H., Nguyen X., Narhi L., Chemmalil L., Towers E., Muzammil S., Gabrielson J., Jiang Y. (2011). Applications of circular dichroism (CD) for structural analysis of proteins: Qualification of near- and far-UV CD for protein higher order structural analysis. J. Pharm. Sci..

[3] Jávorfi T., Hussain R., Myatt D., Siligardi G. (2010). Measuring circular dichroism in a capillary cell using the B23 synchrotron radiation CD beamline at diamond light source. Chirality.

[4] Siligardi G., Hussain R., Owens R.J. (2015). Structural Proteomics: High-Throughput Methods. Methods in Molecular Biology.

[5] Hussain R., Jávorfi T., Rudd T.R., Siligardi G. (2016). High-throughput SRCD using multi-well plates and its applications. Sci. Rep..

[6] Le Vay K., Carter B.M., Watkins D.W., Tang T.-Y.D., Ting V.P., Cölfen H., Rambo R.P., Smith A.J., Anderson J.L.R., Perriman A.W. (2020). Controlling Protein Nanocage Assembly with Hydrostatic Pressure. J. Am. Chem. Soc..

[7] Hussain R., Jávorfi T., Siligardi G. (2021). CD Imaging at High Spatial Resolution at Diamond B23 Beamline: Evolution and Applications. Front. Chem..

[8] Hussain R., Jávorfi T., Siligardi G. (2012). Circular dichroism beamline B23 at the Diamond Light Source. J. Synchrotron Radiat..

[9] Honisch C., Hussain R., Siligardi G., Ruzza P. (2020). Influence of small molecules on the photo-stability of water soluble porcine lens proteins. Chirality.

[10] Hussain R., Longo E., Siligardi G. (2018). UV-Denaturation Assay to Assess Protein Photostability and Ligand-Binding Interactions Using the High Photon Flux of Diamond B23 Beamline for SRCD. Molecules.

[11] Pattison D.I., Rahmanto A.S., Davies M.J. (2012). Photo-oxidation of proteins. Photochem. Photobiol. Sci..

[12] Ruzza P., Honisch C., Hussain R., Siligardi G. (2021). Free Radicals and ROS Induce Protein Denaturation by UV Photostability Assay. Int. J. Mol. Sci..

[13] Kerwin B.A., Remmele R.L. (2007). Protect from light: Photodegradation and protein biologics. J. Pharm. Sci..

[14] Xu G., Chance M.R. (2007). Hydroxyl Radical-Mediated Modification of Proteins as Probes for Structural Proteomics. Chem. Rev..

[15] Crow J.P. (1997). Dichlorodihydrofluorescein and dihydrorhodamine 123 are sensitive indicators of peroxynitrite in vitro: Implications for intracellular measurement of reactive nitrogen and oxygen species. Nitric Oxide.

[16] Drake A.F., Siligardi G., Gibbons W.A. (1988). Reassessment of the electronic circular dichroism criteria for random coil conformations of poly(l-lysine) and the implications for protein folding and denaturation studies. Biophys. Chem..

[17] Siligardi G., Drake A.F. (1995). The importance of extended conformations and, in particular, the PII conformation for the molecular recognition of peptides. Biopolymers.

[18] Provencher S.W., Glöckner J. (1981). Estimation of globular protein secondary structure from circular dichroism. J. Biochem..

[19] Hussain R., Benning K., Jávorfi T., Longo E., Rudd T.R., Pulford B., Siligardi G. (2015). CDApps: Integrated software for experimental planning and data processing at beamline B23, Diamond Light Source. J. Synchrotron Radiat..

[20] Gupta B.S., Taha M., Lee M.J. (2015). Buffers more than buffering agent: Introducing a new class of stabilizers for the protein BSA. Phys. Chem. Chem. Phys. PCCP.

[21] Janc T., Vlachy V., Lukšič M. (2018). Calorimetric studies of interactions between low molecular weight salts and bovine serum albumin in water at pH values below and above the isoionic point. J. Mol. Liq..

[22] Zhao G., Chasteen N.D. (2006). Oxidation of Good’s buffers by hydrogen peroxide. Anal. Biochem..

[23] Baker C.J., Mock N.M., Roberts D.P., Deahl K.L., Schmidt W.F., Kochansky J. (2007). Interference by Mes [2-(4-morpholino) ethanesulfonic acid] and related buffers with phenolic oxidation by peroxidase. J. Free Radic. Biol. Med..

