# Supplementary material for: Free Radical Generation in Far-UV Synchrotron Radiation Circular Dichroism Assays—Protein and Buffer Composition Contribution
Source: Int J Mol Sci. 2021 Oct 20;22(21):11325. doi: 10.3390/ijms222111325 (PMC8583428; doi:10.3390/ijms222111325)
Supplement: Supplementary file 1 [file ijms-22-11325-s001.zip › ijms-1314115-supplementary.pdf]

Free Radical Generation in Far-UV Synchrotron Radiation Circular Dichroism Assays—Protein and Buffer Composition Contribution

Paolo Ruzza [1,*], Claudia Honisch [1,2], Rohanah Hussain [3] and Giuliano Siligardi [3,*]

[1] Institute of Biomolecular Chemistry of CNR, Padua Unit, Via Marzolo, 1, 35131 Padova, Italy; claudiahonisch@gmail.com
[2] Department of Chemical Sciences, University of Padua, Via Marzolo, 1, 35131 Padova, Italy;
[3] Diamond Light Source Ltd., Harwell Science and Innovation Campus, Didcot, Oxfordshire OX11 0DE, UK; rohanah.hussain@diamond.ac.uk
* Correspondence: paolo.ruzza@unipd.it (P.R.); giuliano.siligardi@diamond.ac.uk (G.S.)

SUPPLEMENTARY INFORMATION

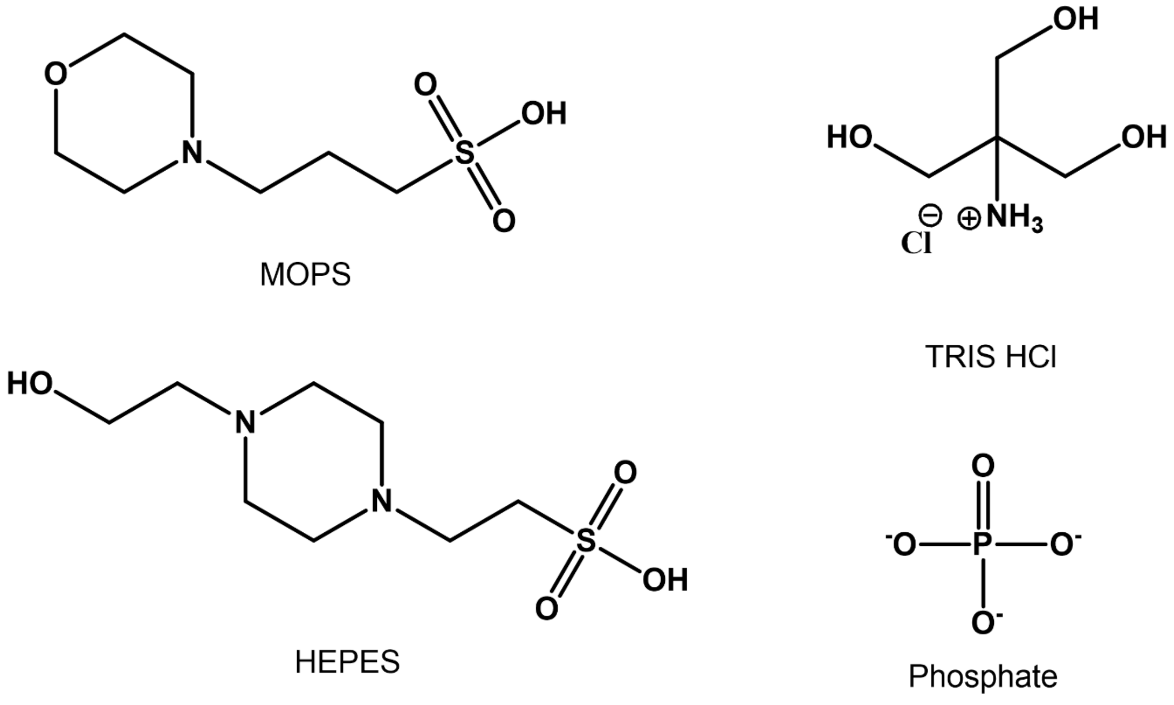

Figure S1. Structure of the investigated buffering agents.

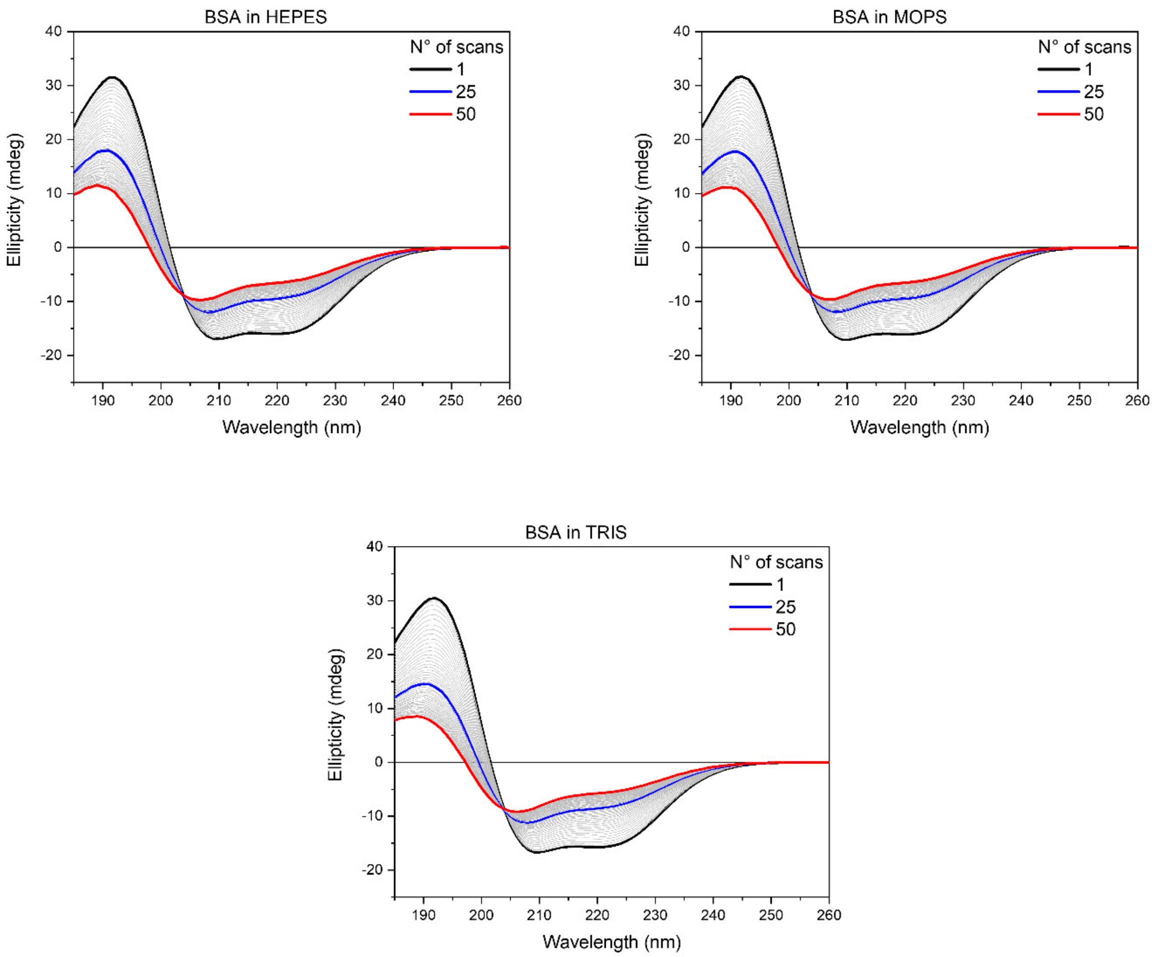

Figure S2. SRCD UV-denaturation experiments performed on Bovine Serum Albumin fatty acid free (BSAffa) in the investigated buffers. 50 consecutive SRCD spectra were acquired at Module B endstation of beamline B23 at Diamond Light Source synchrotron facility, Harwell Science and Innovation Campus (Didcot, UK), using 0.02 cm pathlength quartz cuvettes and 0.5 mg/mL (7.6 µM) protein concentration. Bandwidth was 1 nm, scan speed was 39 nm/min, and top-up mode ring current of 300 mA.

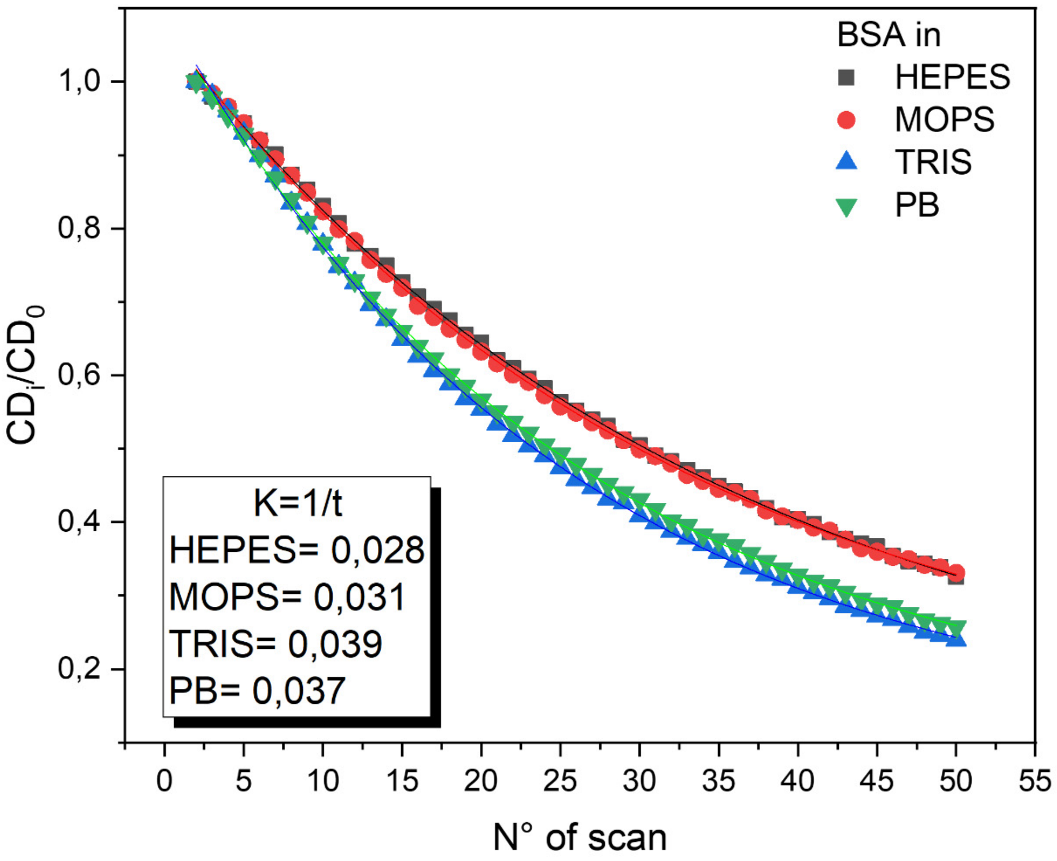

Figure S3. CD_i/CD_0 at 192 nm plotted versus the number of SRCD scans for Bovine Serum Albumin fatty acid free (BSAffa). Data were fitted with ExpDec1 function in OriginPro2018 software (OriginLab Corporation, MA, USA), $y = y_0 + Ae^{(-x/t)}$ where A=amplitude, t=time constant and y_0=offset. The derived parameter rate of denaturation *k=1/t* (min^{-1}) are calculated and shown in the box insert.

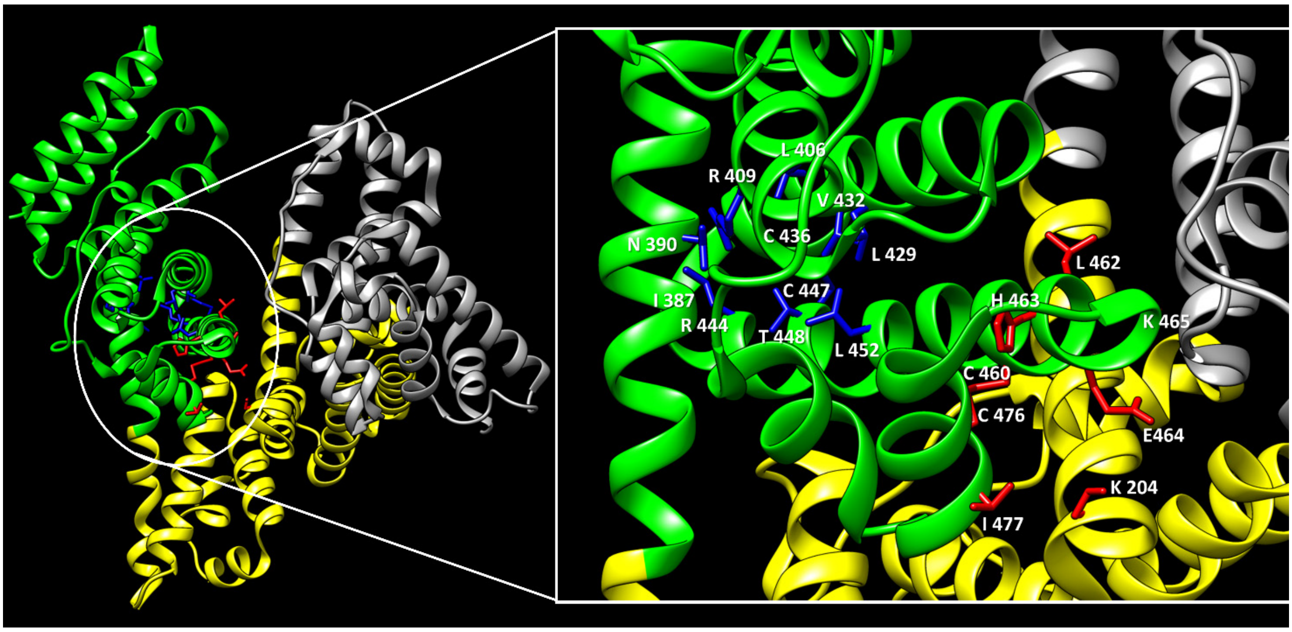

Figure S4. Domain structure of BSA. Domain I in gray; domain II in yellow; domain III in green. In blue the side-chains of residues involved in the interaction with HEPES molecules (Ile 387, Asn 390, Leu 406, Arg 409, Leu 429, Val 432, Cys 436, Arg 444, Cys 447, Thr 448, and Leu 45) and in red the side-chains of residues involved in the interaction with MOPS molecules (Lys 204, Cys 460, Leu 462, His 463, Glu 464, Lys 465, Cys 476, and Thr 477).

Picture originated by UCSF Chimera softwere (Pettersen et al, *J Comput Chem* **2004**; 25(13):1605-12) starting from PDB file: 3V03.

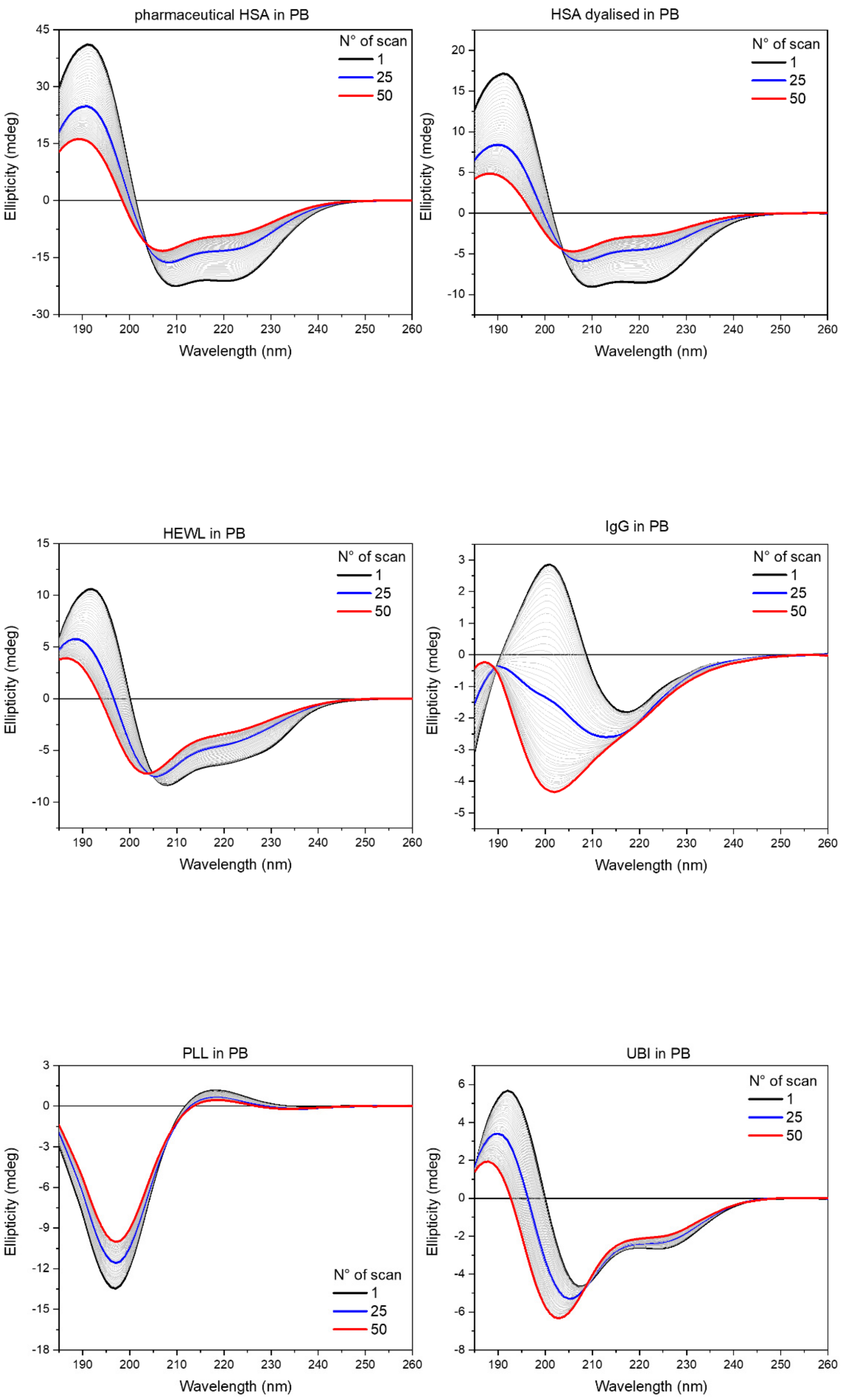

Figure S5. SRCD UV-denaturation experiments performed on Bovine Serum Albumin fatty acid free (BSAffa) pharmaceutical human serum albumin (HSA, 7.5 µM), lysozyme (HEWL, 34.9 µM), ubiquitin (Ubi, 58.1 µM), rabbit IgG (IgG, 3.5 µM) and poly-L-lysine (PLL, 125.0 µM), each 0.5 mg/mL in phosphate buffer. 50 consecutive SRCD spectra were acquired at Module B endstation of beamline B23 at Diamond Light Source synchrotron facility, Harwell Science and Innovation Campus (Didcot, UK), using 0.02 cm pathlength quartz cuvettes. Bandwidth was 1 nm, scan speed was 39 nm/min, and top-up mode ring current of 300 mA.

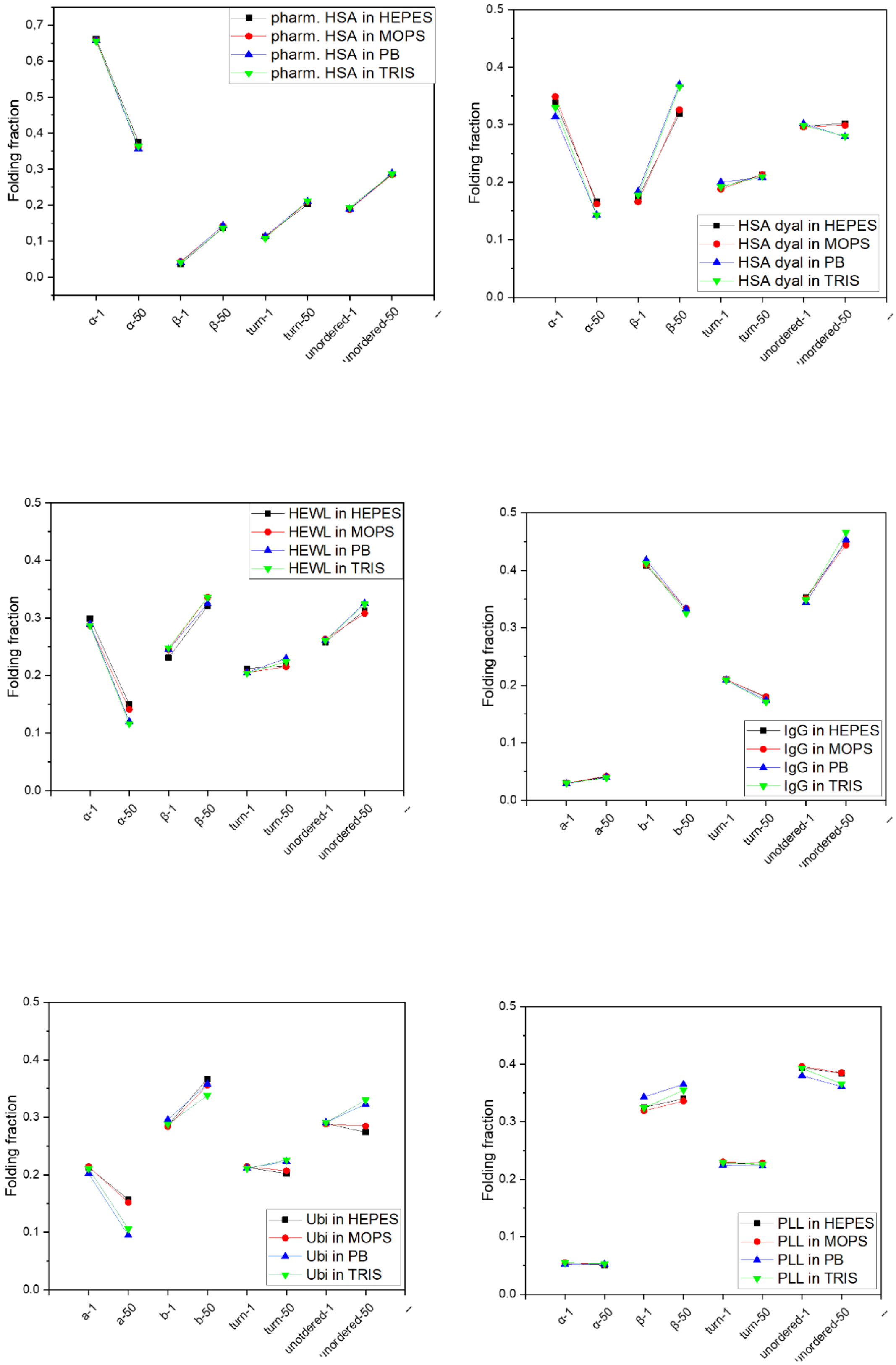

Figure S6. Change in secondary structure content determined for Bovine Serum Albumin fatty acid free (BSAffa) pharmaceutical human serum albumin (HSA), lysozyme (HEWL), ubiquitin (Ubi), rabbit IgG (IgG) and poly-L-lysine (PLL) in all the investigated buffers after 50 consecutive SRCD spectra were collected. The calculations were performed using CDApps software (Hussain et al, *J Synchrotron Radiat* **2015**, 22, 1-11) with CONTINLL algotithm.

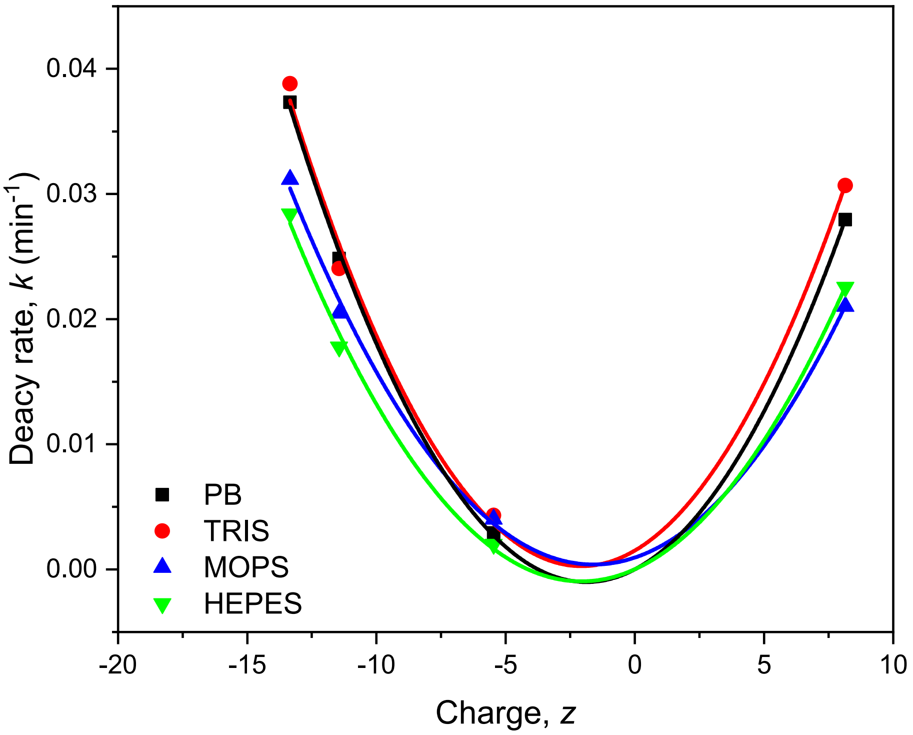

Figure S7. Correlation between protein charge (z) and decay rate values of protein UV-denaturation.

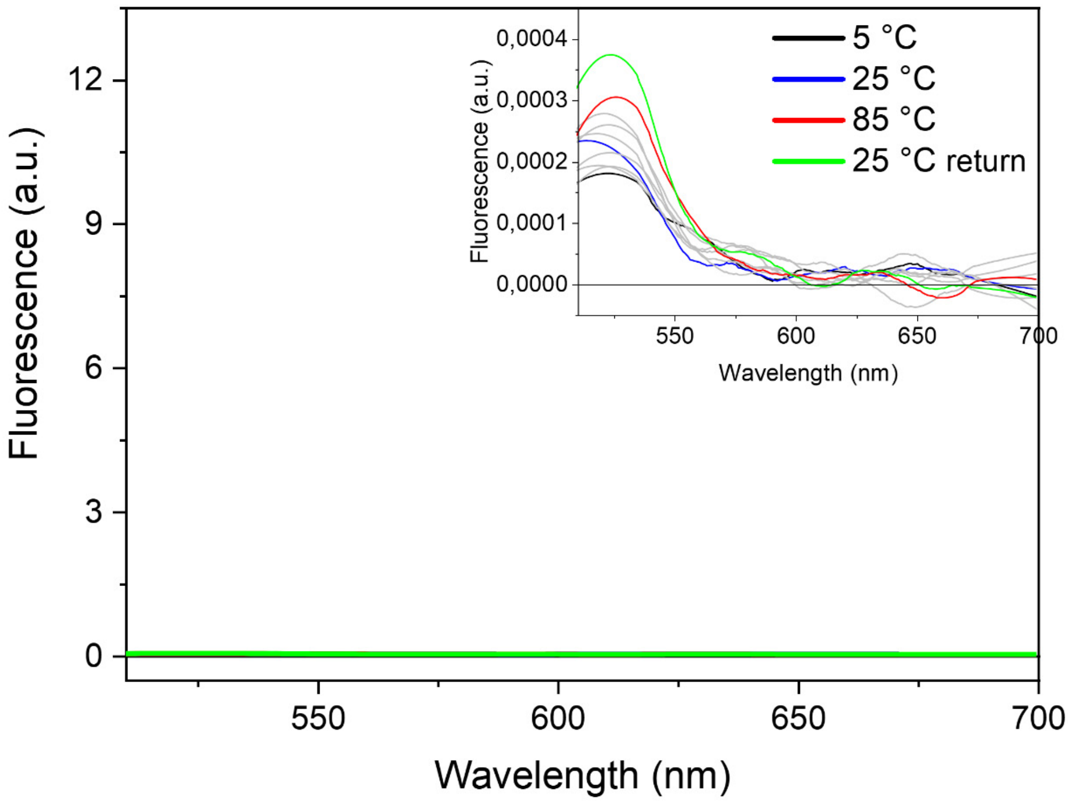

Figure S8. Fluorescence emission spectra of DHR-123 when heated from 5 to 85°C. Briefly, 1.5 µL of DHR-123 in DMSO (2 mg/mL) were added to 3000 µL of 10 mM PB, pH 7.4, in a Suprasil fluorescence cell with 1.0 cm path length, emission spectra were collected with 10°C steps, allowing 8 min equilibration, using a Chirascan Plus CD Spectropolarimeter with fluorescence attachment (Applied Photophysics, Leatherhead, UK).

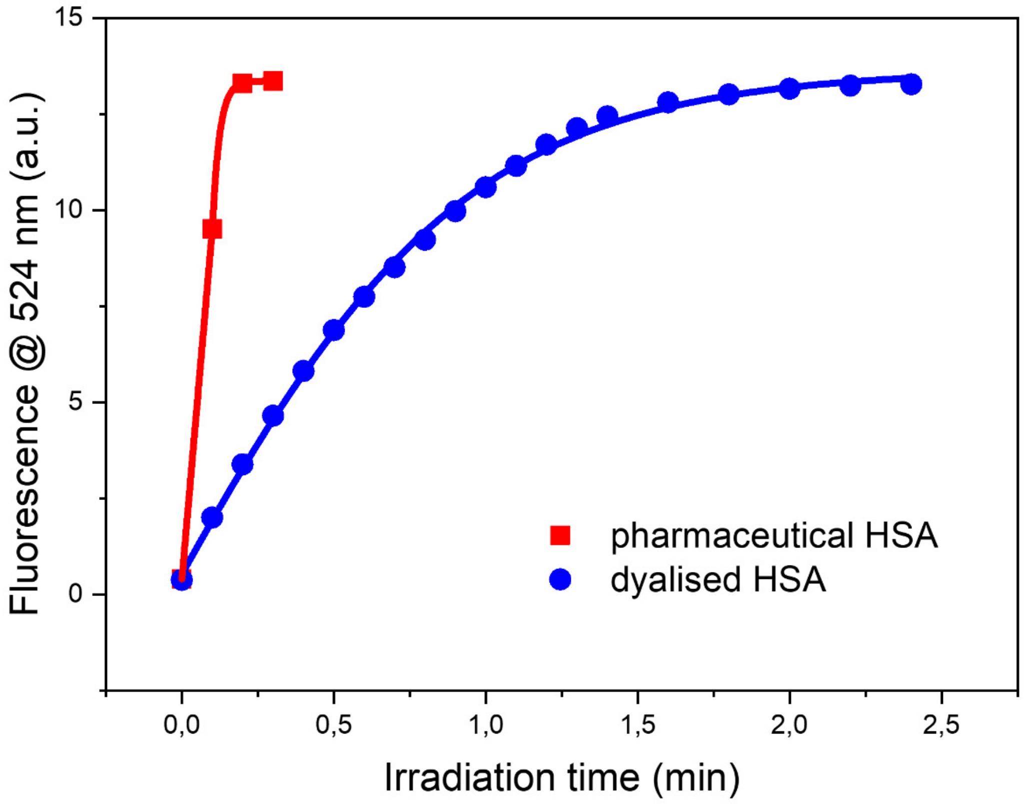

Figure S9. Fluorescence emission spectra of oxidised DHR-123 in presence of pharmaceutical Human Serum Albumin (HSA) or its dyalised solution, obtained to remove stabiliser excess. 1.5 µL of DHR-123 in DMSO (2 mg/mL) were added to 3000 µL 0.1 mg/mL equivalent to 1.5 µM of HSA or dyalised HSA solutions in 10 mM PB, pH 7.4, in a Suprasil fluorescence cell with 1.0 cm pathlength and irradiated using a BioLink 254 photoreactor (Vilber, Eberhardzell, Germany). The fluorescence emission spectrum of irradiated DHR-123 solution in the 510-700 nm range (Ex 505 nm, slit 4 nm) was recorded at different times.

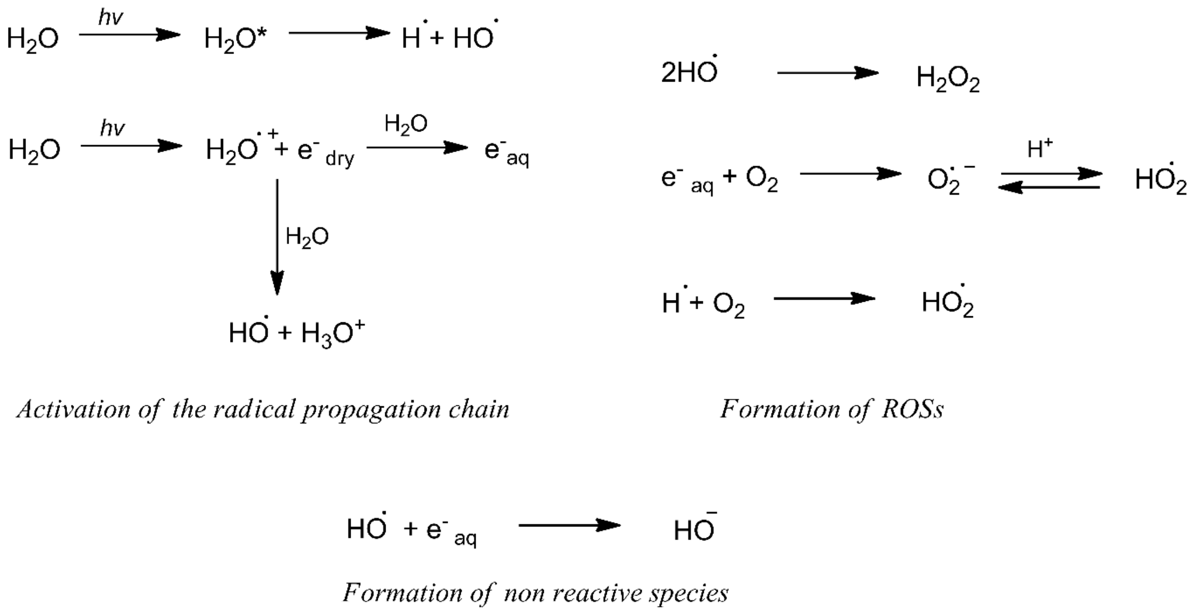

Scheme S1. UV irradiation effects on water molecules.

H_2O^*: excited water molecule; e^-_{aq}: hydrated electron; e^-_{dry}: dry electron.

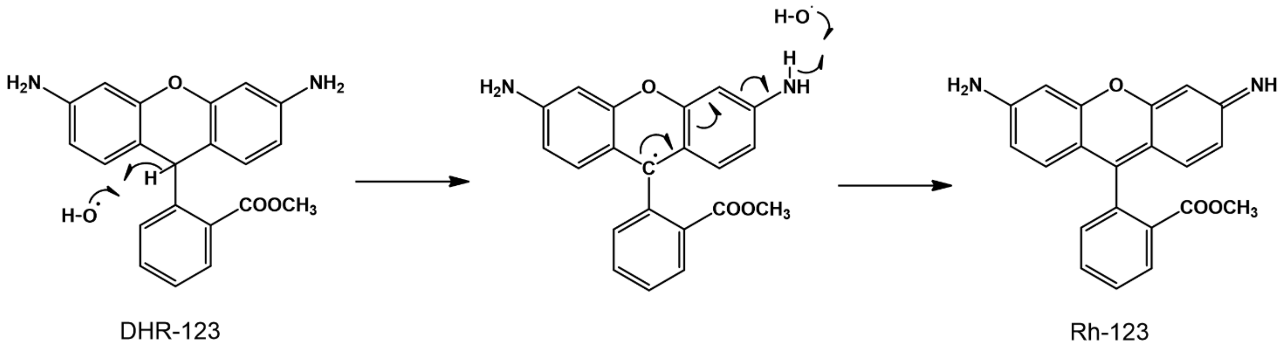

Scheme S2. Dihydrorhodamine-123 (DHR-123) oxidation.

Buffer Pair	p-value					
	HEWL	IgG	HSA	BSA	Ubiquitin	PLL
PB/Tris	3.99E-21	0.01422	1.80E-08	2.30E-31	1.21E-19	2.15E-57
PB/MOPS	1.59E-48	4.48E-12	8.77E-63	2.22E-78	6.48E-15	6.30E-34
PB/HEPES	6.18E-35	2.51E-31	1.11E-72	2.05E-115	1.28E-11	4.74E-01
Tris/MOPS	1.79E-62	4.66E-16	1.61E-59	7.44E-86	1.81E-06	2.36E-30
Tris/HEPES	4.77E-52	3.68E-34	7.15E-71	3.35E-116	6.59E-32	4.47E-79
MOPS/HEPES	4.67E-11	7.74E-08	1.18E-29	8.11E-91	1.94E-33	5.91E-45

Table S1. Statistics Tests. Hypothesis testing were carried out using two sample t-test with k and its associated standard deviation values as the summarised data using OriginLab software on all proteins tabulated in Table 1 and with all possible buffer pairs. The statistical tests showed all results to be significantly different (p-value < 0.05) with the exception of PLL comparison between PB and HEPES (p-value 0.474).
